# Anxiety and depression in young people in the UK: demographic differences and growing treatment gaps

**DOI:** 10.1192/j.eurpsy.2025.148

**Published:** 2025-08-26

**Authors:** J. Dykxhoorn

**Affiliations:** Division of Psychiatry, UCL, London, United Kingdom

## Abstract

**Background:**

Rates of common mental disorders (CMD), such as anxiety and depression, treated in primary care have increased among young adults in the UK. However, it remains unclear whether this increase reflects a greater tendency to seek help for CMD or a rise in CMD symptoms over time. Additionally, it is not clear if these increases are more pronounced in specific sociodemographic groups. This research examined the temporal trends of primary care-recorded CMD and self-reported CMD symptoms in young adults.

**Methods:**

We included participants born between 1980 and 2003 in two datasets: UK primary care records from the *Clinical Practice Research Datalink*, and longitudinal cohort data from *Understanding Society.* We estimated the annual incidence of primary care-recorded CMD overall and by sex, age, birth cohort, ethnicity, country, region, and deprivation from 2009 to 2019, and explored changes over time using incidence rate ratios. We compared these trends to annual estimates of self-reported CMD symptoms from longitudinal cohort data between 2009-10 and 2019-20, calculating ratios to explore changes in CMD symptoms over time by sociodemographic group.

**Results:**

Between 2009 and 2019, the incidence of recorded CMD increased by 9·90% and CMD symptoms increased by 19·33%. The sharpest increases for both recorded CMD and CMD symptoms were observed in older adolescents (ages 16-19) and those born after 1995. Recorded CMD increased more in males (20·61%, increase) than in females (7·65%), despite similar increases in CMD symptoms. Recorded CMD increased the most in the least deprived areas of England (16·34%) compared to the most deprived areas (3·55%), despite similar increases in CMD symptoms across all levels of deprivation.

**Conclusions:**

Both primary care-recorded CMD and self-reported CMD symptoms in young adults increased between 2009-19, suggesting that the rising rates of CMD treated in primary care may reflect heightened symptoms of CMD. However, differences in the patterns of recorded CMD and CMD symptoms across sociodemographic groups highlight potential misalignment between mental health care provision and underlying population need, suggesting that the groups with the highest burden of CMD symptoms may not be the groups most likely to receive care.

**Disclosure of Interest:**

None Declared

